# A Laboratory Goniometer System for Measuring Reflectance and Emittance Anisotropy

**DOI:** 10.3390/s121217358

**Published:** 2012-12-13

**Authors:** Peter P. J. Roosjen, Jan G. P. W. Clevers, Harm M. Bartholomeus, Michael E. Schaepman, Gabriela Schaepman-Strub, Henk Jalink, Rob van der Schoor, Arjan de Jong

**Affiliations:** 1 Laboratory of Geo-Information Science and Remote Sensing, Wageningen University, P.O. Box 47, 6700 AA Wageningen, The Netherlands; E-Mails: peter.roosjen@wur.nl (P.P.J.R.); harm.bartholomeus@wur.nl (H.M.B.); 2 Remote Sensing Laboratories, Department of Geography, University of Zurich, Winterthurerstrasse 190, CH-8057 Zurich, Switzerland; E-Mail: michael.schaepman@geo.uzh.ch; 3 Institute of Evolutionary Biology and Environmental Studies, University of Zurich, Winterthurerstrasse 190, CH-8057 Zurich, Switzerland; E-Mail: gabriela.schaepman@ieu.uzh.ch; 4 Wageningen UR Greenhouse Horticulture, P.O. Box 644, 6700 AP Wageningen, The Netherlands; E-Mails: henk.jalink@wur.nl (H.J.); rob.vanderschoor@wur.nl (R.S.); 5 Alterra, Wageningen University & Research Centre, P.O. Box 47, 6700 AA Wageningen, The Netherlands; E-Mail: arjan2.dejong@wur.nl

**Keywords:** reflectance anisotropy, BRDF, emittance anisotropy, laboratory goniometer, biophysical parameter retrieval

## Abstract

In this paper, a laboratory goniometer system for performing multi-angular measurements under controlled illumination conditions is described. A commercially available robotic arm enables the acquisition of a large number of measurements over the full hemisphere within a short time span making it much faster than other goniometers. In addition, the presented set-up enables assessment of anisotropic reflectance and emittance behaviour of soils, leaves and small canopies. Mounting a spectrometer enables acquisition of either hemispherical measurements or measurements in the horizontal plane. Mounting a thermal camera allows directional observations of the thermal emittance. This paper also presents three showcases of these different measurement set-ups in order to illustrate its possibilities. Finally, suggestions for applying this instrument and for future research directions are given, including linking the measured reflectance anisotropy with physically-based anisotropy models on the one hand and combining them with field goniometry measurements for joint analysis with remote sensing data on the other hand. The speed and flexibility of the system offer a large added value to the existing pool of laboratory goniometers.

## 1. Introduction

Earth observation in the reflective solar domain can provide a number of key biophysical and biochemical products of vegetation, such as the fraction of absorbed photosynthetically active radiation, leaf area index, canopy structure, chlorophyll content and water content. The first two have been identified as essential climate variables (ECVs) by the Global Climate Observing System (GCOS) Steering Committee [1] and are key variables that are both used in surface process models and retrieved from remote sensing observations in the reflective solar domain. Various algorithms are used to derive these products, but they still mostly relate to nadir or directionally normalized observations [2]. There are only a few products that rely on estimates of the bidirectional reflectance distribution function (BRDF), for instance products from NASA’s Moderate Resolution Imaging Spectrometer (MODIS) and Multi-angle Imaging SpectroRadiometer (MISR) [3,4].

For operational Earth observation applications multisensor data usage will be required to increase the number of observations within a given time period, particularly relevant in regions with frequent cloud cover [5]. This will result in an increase of high-quality data in time-series for monitoring activities. However, instability of retrieval algorithms to directional effects will degrade the accuracy of derived products. As a result, information on the BRDF of vegetation and soils is relevant for normalizing images taken under different illumination and/or viewing conditions [2], but on the other hand it has been shown that multi-angular observations provide additional information that can be used to improve the accuracy of retrieved products [5–7].

The BRDF of surface targets contains information on structure and composition that cannot be inferred from spectral properties alone [8]. The BRDF as defined by [9] is the ratio of the radiance reflected in a specific direction to the incident irradiance for a given illumination-viewing geometry and wavelength. The BRDF can be modelled by performing BRF (bidirectional reflectance factor) measurements at different viewing and illumination angles [10]. The BRF is the ratio of the radiance reflected from a target to the radiance from a lossless isotropic (Lambertian) surface for a given illumination-viewing geometry and wavelength. When using, for instance, a Spectralon reference panel instead of a lossless surface, this ratio has to be multiplied with the reflectance of the reference panel at the same illumination-viewing geometry and wavelength.

Modelling the BRDF of targets using sampled BRF measurements can be done either in an empirical way or through physically-based models. Most research until now faces the problem of too few directional sampling combinations for optimally fitting a BRDF model. Multidirectional reflectance measurements acquired under controlled laboratory conditions can solve this problem because a large amount of directions can be sampled. Examples of such a laboratory set-up are the European Goniometric Facility (EGO) of the Joint Research Centre [11], the Field Goniometer System (FIGOS) of the University of Zurich that can also be used in the laboratory [12], the Sandmeier Field Goniometer (SFG) of NASA Ames, which is based upon and nearly identical to the FIGOS design [13], the Israeli Goniometric Facility (IGF) of the University of Tel-Aviv [14], the University of Lethbridge Goniometer System (ULGS) [15] and the Compact Laboratory Spectro-Goniometer (CLabSpeG) of the Catholic University Leuven [16]. Some of these can also be applied in the field under natural conditions, but all systems are (a) limited in the flexibility of the system in terms of hemisphere size and measurement positions, and (b) taking a long time for sampling a full hemisphere, which causes problems for plants susceptible to changes in leaf angle and orientation due to changes in turgor in the plant tissue and changes in soil due to dehydration. All these goniometers measure reflectance anisotropy; we are aware of only one system that can also measure the anisotropy of thermal emittance [17]. However, this system is designed for field measurements and the azimuth angle has to be set by hand.

We present a laboratory goniometer facility that is designed to assess the anisotropic reflectance behaviour of soils, leaves and small canopies under controlled illumination conditions. Advantages are the high measurement speed and the possibility of adjusting measurement distance depending on zenith/azimuth angle. In addition to a spectroradiometer, it is also able to carry a thermal camera. This laboratory goniometer system has been realized by Wageningen University (see Section 2).

In the following sections we first describe the system. Next, three showcases with some first results are presented. As first case, the capacity to get fast reflectance measurements over the full hemisphere is illustrated. Subsequently, the flexibility of the system in terms of measurement set-up for doing image simulation by performing measurements in the horizontal plane is shown. Finally, mounting another sensor is illustrated by showing results for the thermal anisotropy of a vegetation target. At the end of this paper an outlook is presented with respect to BRDF modeling and the upscaling to a remote sensing level.

## 2. The Laboratory Goniometer System

**Figure 1.**
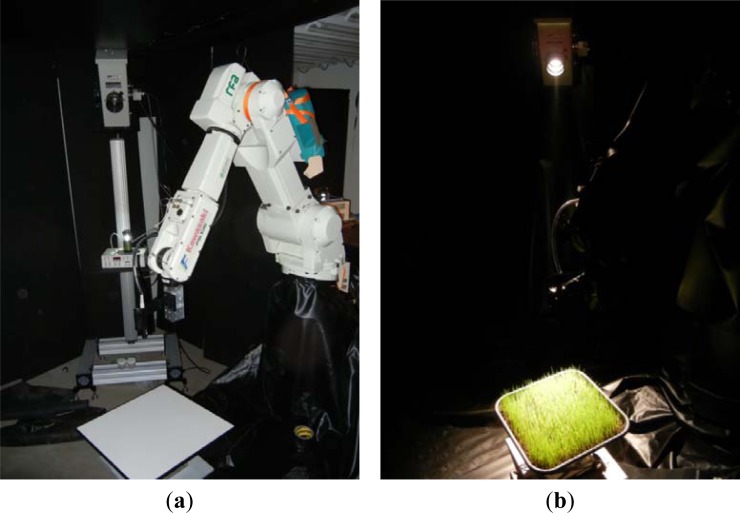
(**a**) Laboratory set-up with a Spectralon reference panel before covering the instruments with black materials; (**b**) laboratory set-up during the measurements: the walls are covered with panels that are painted with black latex and the floor, ceiling and the robot are covered with black PVC foil.

The core of the goniometer system is an industrial robot arm, on which an ASD FieldSpec 3 spectroradiometer and/or a NEC thermal camera can be mounted (Figure 1). In the following subsections the components of the system will be described.

### 2.1. Kawasaki Robot

The robotic system consists of a Kawasaki FS10E industrial robot, which can be programmed to reach every point within a 155 cm working radius due to the presence of six movement axes. It has a lifting capacity of 10 kg, which is enough to support simultaneous mounting of both a spectrometer and thermal camera. The position repeatability is ±0.1 mm, which is of major importance to obtain reliable repetitive measurements. The use of a robotic positioning system was also recommended before [12], because it is fast and highly reproducible. On average, taking a measurement and moving to the next position takes about 10 seconds. As a result, the target can be assumed constant while sampling a full hemisphere, which is an advantage compared to most other existing laboratory goniometers.

In order to be used as a goniometer, the robot arm can be fully programmed in two modes: the “hemisphere mode” for hemispherical measurements and the “scan mode” for measurements in the horizontal plane. In the hemisphere mode there are a number of options, where the most common set-up is to measure view zenith angles from −90° to +90° off nadir. In this set-up the radius of the hemisphere can be varied from 25 cm up to 100 cm, although some positions cannot be reached at larger hemisphere sizes. Theoretically, one can measure an unlimited number of positions of the hemisphere. Positions are programmed in such a way that we also can take measurements close to the hot-spot, making sure that the robot itself is not in between the illumination source and the target (avoiding shadow casting by the instrument itself). The hot-spot position refers to the position where illumination and observation direction coincide precisely.

The flexibility of the robot system even allows the positioning of the sensor below the horizontal plane, thus allowing measurement of the amount of light passing through an object. In the scan mode the sensor is moved in the horizontal plane, where grid spacing and height above the surface are (even per point) programmable.

### 2.2. Illumination

A 1,000 W Quartz Tungsten Halogen (QTH) lamp is used as artificial light source, with focused output beam [18]. QTH lamps are visible and near-infrared light sources used in reflectance spectroscopy because of their smooth spectral curve and stable output. In the near future we will study the need of further illumination improvement by using a collimator.

### 2.3. Sensors

Spectral reflectance measurements are performed with an ASD Fieldspec 3 spectroradiometer. It performs reflectance and radiance measurements in the range of 350–2,500 nm. The spectral sampling interval is 1.4 nm at 350–1,000 nm, and 2 nm at 1,000–2,500 nm. The instantaneous field of view (IFOV) of the instrument can be selected as 1°, 8 or 25° using fore-optics. The hemisphere can be sampled continuously up to a few degrees from the hot-spot position. Since both the spectroradiometer and the illumination source have a conical field of view/illumination, we actually measure a biconical reflectance factor [19]. The steering of the robotic arm and the reading of the spectroradiometer are fully automated in such a way that biconical reflectance measurements are obtained at a fixed, predefined distance from the target at predefined positions (viewing angles).

In addition to reflectance measurements, thermal emittance can be measured with a NEC TH9100 Infrared Thermal imager. It is a thermal-infrared imaging camera, operating monospectrally in the 8–14 μm region. The camera has a temperature resolution of 0.02 K (with signal-to-noise improvement) and with an IFOV of 1.2 mrad it creates images of 320 × 240 pixels. So, in addition to the reflectance anisotropy, also the thermal anisotropy properties can be measured.

### 2.4. Software

**Figure 2.**
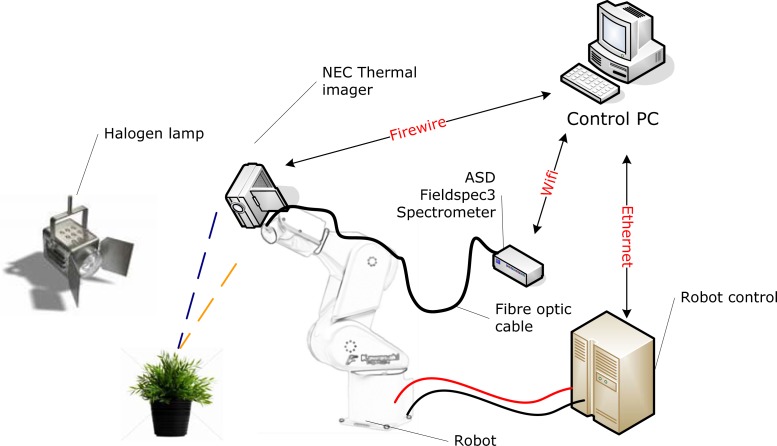
Topology of the goniometer facility.

The total set-up is controlled by the “Control PC” with custom built software. A regular desktop computer or laptop can be used, as long as the requirements for connection options are met. The equipment is connected with the Control PC (Figure 2) through Ethernet (Robot), Wifi (Spectrometer) and Firewire (Thermal camera).

On the Control PC a project directory is created where all information about the measurement is stored, including configuration settings, calibration parameters and the measurement results. Various wizards provide measurement options: the instrument selection, the performance of required calibrations and the selection of hemispherical or plane measurement coordinates. The measurement settings can be saved to an XML file, or imported from previously saved or edited XML files. When a position is reached during the measurement sequence, the measurements are performed and directly stored. In this way, there is no loss of data if the process has to be aborted during the measurement of the full hemisphere or plane. The exact time and position of each individual measurement are stored in the file name, while the raw data are stored in separate text files. These text files contain the wavelength, digital number, radiance and reflectance for the spectral measurements and absolute temperature values for the thermal measurements.

### 2.5. Measurement Environment

To avoid unwanted scattering, the walls of the laboratory are covered with wall panels that are painted with black latex. The robot, the floor and the ceiling are covered with black PVC foil as can be seen in Figure 1(b). Both the latex and the foil have a reflectance of less than 3% in the visible and NIR.

## 3. Results

In this section we will present three showcases of first results obtained with the laboratory goniometer system. In Section 3.1 attention is paid to hemispherical reflectance measurements. Since this was the main goal of this goniometer set-up, most attention is paid to these measurements. These anisotropy measurements were also used to test the performance of the system. In Section 3.2 an example is given showing measurements performed in the horizontal plane. Finally, Section 3.3 illustrates the showcase of obtaining the thermal anisotropy of vegetation.

### 3.1. Hemisphere Mode and System Performance

40 × 40 cm^2^ plots with 18 days old lawn grass (*Lolium perenne* L.) and watercress (*Nasturtium officinale* R.Br.) served as the objects for the biconical reflectance measurements. These targets were chosen for their different leaf angle distribution (LAD), because it is expected that this structural property has a major effect on reflectance anisotropy. Lawn grass has an erectophile LAD and watercress has a planophile LAD. Soil background was a dark mull soil with a 17% organic matter content.

**Figure 3.**
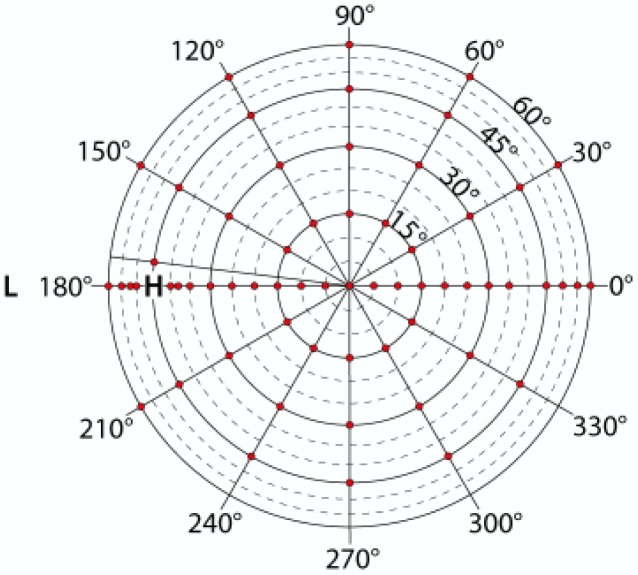
Measurement positions with an illumination zenith angle of 45°. L is the position of the light source and H is the hot-spot.

For the measurements, the sensor–target distance was set at 40 cm. The IFOV of the ASD was set at 8°. Measurements on the lawn grass and watercress were taken at an illumination zenith angle of 45°. The targets were measured at a resolution of 30° over the full azimuthal plane and of 15° for zenith angles from −60° to +60°. Each scan started and ended with a series of measurements over the principal plane with a zenith angle resolution of 5°. Around the hot-spot additional measurements were taken. Azimuth angles of 240°, 270°, 300° and 330° at a zenith angle of 60° could not be measured because the robotic arm was casting shadow on the target at those positions. Per scan, 96 measurements were taken at 62 different positions (Figure 3). Before and after each scan, a Spectralon panel was measured from nadir for calculating (biconical) reflectance factors.

**Figure 4.**
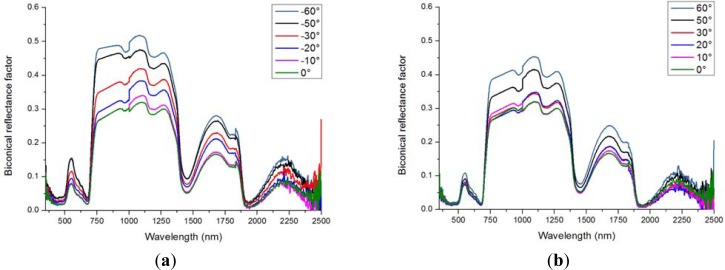
Lawn grass reflectance at a 10°, 20°, 30°, 50° and 60° viewing zenith angle in the principal plane at an illumination angle of 45°: (**a**) is the 18 days old lawn grass in the backward scatter direction; and (**b**) is the 18 days old lawn grass in the forward scatter direction. In both graphs a measurement from the nadir position (zenith angle is 0°) is included as a reference.

In Figure 4 the spectral signatures of lawn grass are shown for different viewing angles. For clarity, these measurements are separated into two graphs, showing the spectral signatures in backward scatter direction (Figure 4(a)) and in forward direction (Figure 4(b)). Lowest reflectance occurred closest to nadir, because at this position most of the dark soil background was visible. With increasing off-nadir viewing angle more leaf layers were visible, yielding an increase in the measured reflectance factor. As expected, close to the hot-spot (Figure 4(a)) the highest reflectance values were obtained. For the watercress the highest reflectance was also found close to the hot-spot (results not shown), but differences between the distinctive angles were less than the differences found for the grass. This can be explained by the more erectophile LAD for grass, giving more internal shadow casting within the canopy. This shadow is not observed when looking at the hot-spot position, yielding highest reflectances at that position.

**Figure 5.**
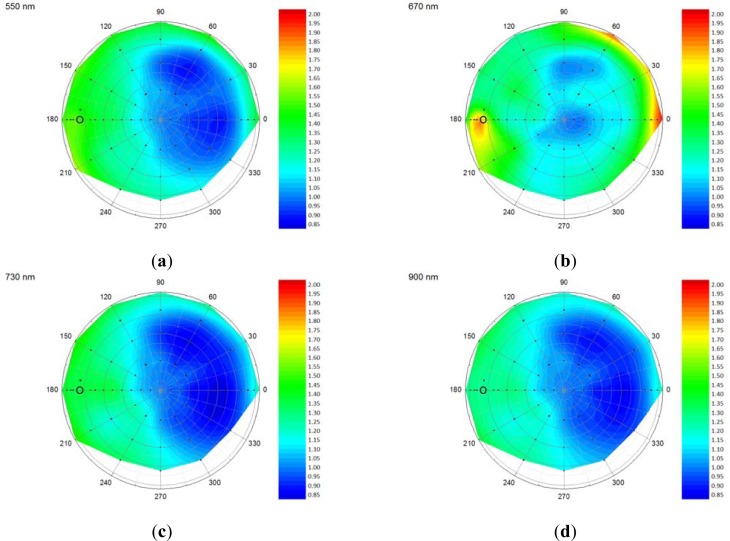

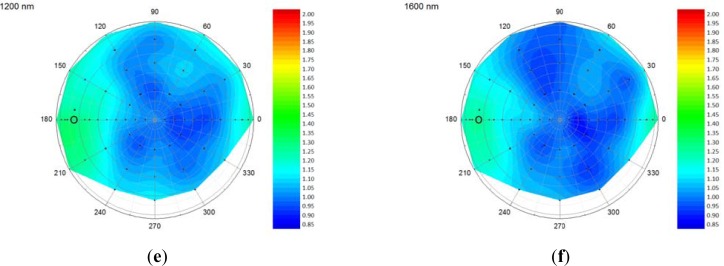
Polar plots of the anisotropy factor of 18 days old watercress at 550 nm (**a**), 670 nm (**b**), 730 nm (**c**), 900 nm (**d**), 1,200 nm (**e**), and 1,600 nm (**f**). The black dots indicate the measurement positions and the circle indicates the hot-spot position (illumination angle is 45°).

Figure 5 displays interpolated polar plots of the anisotropy factor (ANIF) of 18 days old watercress. The ANIF is the nadir normalized reflectance factor [11]. The highest ANIF values were found around the hot-spot position, although the hot-spot was not always that obvious, which probably can be explained by the measurement of biconical reflectances instead of bidirectional reflectances. The biconical measurement set-up integrates over a range of angles, not solely limited to the hot-spot direction only, yielding a blurred effect of the hot-spot. The most pronounced hot-spot effect occurred at 670 nm (Figure 5(b)). In the visible domain, the anisotropic effects were most pronounced and at increasing wavelengths the anisotropic effect diminished. This can be explained by the fact that in the near-infrared part of the spectrum multiple scattering is dominant. Multiple scattering within the canopy causes radiation to be scattered more evenly in all directions which diminishes anisotropic effects [11]. For 550 nm, 730 nm and 900 nm, the lowest ANIF values were found around the dark-spot position. At 670 nm, 1,200 nm and 1,600 nm, the lowest ANIF values were found near the nadir position.

**Figure 6.**
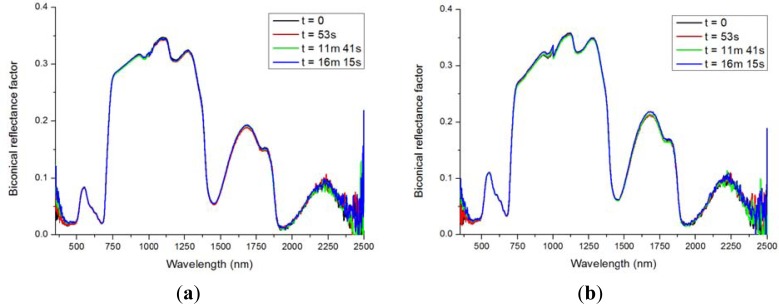
Repetitive measurements at nadir position of (**a**) 18 days old lawn grass; (**b**) 18 days old watercress (t = time after start of hemispherical measurements).

During the acquisition of the measurements over the full hemisphere, the robot was programmed to measure the nadir position four times in order to determine if the reflected signal had changed due to vegetation stress caused by the irradiance of the light source. The results of these measurements are shown in Figure 6. There are no clearly observable differences between the repetitive measurements at nadir position. The average root mean square (RMS) difference with the measurement at t = 0 over the interval 500–1,900 nm is given in Table 1. The low RMS values indicate that there are only minor differences between the repetitive measurements. Therefore, it can be concluded that within the short acquisition time of the measurements there are no signs of dehydration and wilting of the vegetation.

### 3.2. Scan Mode (Horizontal Plane)

In this example pine tree (*Pinus sylvestris* L.) seedlings of about 40 cm height were used. Nine trees were positioned 30 cm apart in a 3 × 3 grid. The seedlings had one dominant shoot that is irregularly shaped. Due to numerous side branches, an irregular canopy pattern was created. The sensor-target distance was set at 40 cm and the IFOV of the ASD was set at 8°. This resulted in a pixel size of 5.6 cm. Measurements of a 50 × 50 cm^2^ area were acquired in scan mode, with a grid spacing of 5 cm. So, there was a small overlap (about 12%) between pixels. An ASD halogen lamp was mounted nadir positioned on the robotic arm to ensure equal illumination of the target at all locations. The area that can be measured in this way is much larger than the area that is uniformly illuminated by the QTH lamp when fixed in one position.

**Figure 7.**
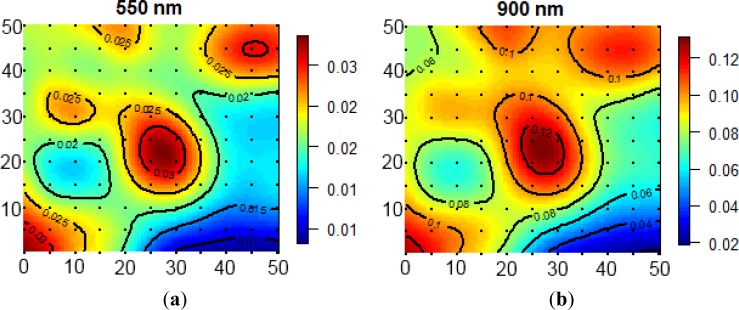
Interpolated biconical reflectance measurements of a 50 × 50 cm^2^ area covered with small pine trees at (**a**) 550 nm, and (**b**) 900 nm (illumination angle close to nadir, constant view zenith angle = 0° in scan mode).

Figure 7 shows interpolated surface reflectance measurements at two wavelengths as an example, namely 550 nm (in the green) and 900 nm (in the near-infrared). The black dots indicate the positions at which the measurements were taken. Due to the dark soil background the upper part of the main shoot showed up as bright spots in the figures. This applied both to the green and near-infrared wavelengths. Peak reflectance was about 0.03 in the green, whereas it was about 0.12 in the near-infrared. The low reflectance in the near-infrared can be explained by the fact that we were dealing with a sparse canopy (and thus measuring a great proportion of the dark soil). However, these measurements show that the system in scan mode is very well able to capture the spatial heterogeneity of this target. The total image cube contains 2,150 spectral bands, although the shortwave infrared wavelengths produced noisy results with the described illumination.

### 3.3. Thermal Anisotropy

As stated before, thermal emittance measurements can be performed in addition to reflectance measurements. In this example the thermal anisotropy of a fully grown grass (*Lolium perenne* L.) canopy has been measured with the thermal camera (Section 2.3). In combination with a sensor-target distance of 40 cm the IFOV of 1.2 mrad yielded a pixel size at nadir of about 0.5 mm. Subsequently thermal images were acquired at all combinations of azimuth angles in steps of 30° and zenith angles in steps of 15° (from −60° to +60°). For this experiment the 1,000 W halogen lamp was positioned at 45° zenith angle.

**Figure 8.**
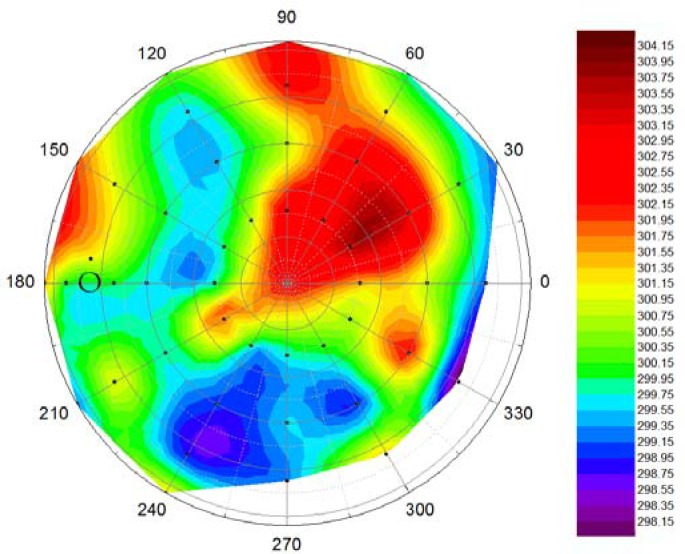
Polar plot of the thermal anisotropy of a grass canopy. The circle indicates the hot-spot position. Temperature is given in Kelvin.

Figure 8 shows an example of a thermal emittance polar plot. The temperature values presented are calculated as the average temperature of the center 25 × 25 pixels of each acquired image. This matches an area of about 1.2 cm by 1.2 cm. The maximum range in observed surface temperatures is about 6 K, which is not extremely large. This can be explained by the dense and homogeneous crop used. Timmermans *et al*. observed a similar range for grass in the field [17]. They found much larger directional effects for other crops. It is expected that temperature differences are related to shadow effects in the canopy, but this still has to be studied further.

## 4. Conclusion and Discussion

In this paper, a laboratory goniometer system formed by a robot, for performing multi-angular measurements has been described. The presented set-up enables acquisition of multi-angular reflectance and thermal emittance measurements of soil, leaves and small canopies. The main advantage of this system is that it is much faster than other goniometers as well as can be equipped with a multitude of sensors. Moreover, the use of a robotic arm results in a flexible system that can easily be adapted for different measurement set-ups, for example allowing measurements below the canopy and measurements in the horizontal plane, as well as testing range dependent anisotropy for scaling issues. Finally, mounting of a thermal camera allows directional observations of the thermal emittance. In this paper, the first results of a variety of measurement possibilities of the goniometer facility are shown with the emphasis on reflectance anisotropy, which is a major research item for the coming years. It seems important that a BRDF library [20] is created with angular measurements of the main agricultural crops, various soil types with varying roughness and small tree canopies, which then should be made available to the scientific community.

Multi-angular laboratory measurements can be used for exploration of the BRDF effect and, subsequently, for validation of BRDF models [21,22]. Most research until now faces the problem of too few directional sampling combinations for fitting a BRDF model. Multidirectional reflectance measurements acquired under controlled laboratory conditions can solve this problem because a large amount of directions can be sampled. Modelling the BRDF of targets using sampled biconical reflectance measurements can be done either in an empirical way or through physically-based models. Empirical and physical models can be combined in semi-empirical (hybrid) models, which are more versatile. Semi-empirical models are often a linear combination of three terms consisting of isotropic, surface and volumetric functions [23,24]. These terms are called kernels and therefore these models are called kernel-driven or kernel-based models [25]. Inversion of such additive models is relatively straightforward. Since linear semi-empirical models have difficulties to describe the complex BRDF of vegetation at very high IFOVs, multiplicative semi-empirical models such as the Rahman-Pinty-Verstraete (RPV) model [26] might be used. The latter models require iterative inversion but this is relatively straight forward and implemented already [27]. Physical RT models rely on physical principles describing the interaction of solar radiation with a target, also including the directional effects. PROSAIL has become one of the most popular physical RT models due to its ease of use, robustness and consistent validation results [28]. PROSAIL or the extended soil-leaf-canopy (SLC) version [29] can simulate directionality of observations, but they are still rather simple in that they represent the canopy as a horizontally homogeneous turbid medium. More complex 3D radiative transfer models such as DART [30] are particularly interesting for simulating BRDF properties. Combining semi-empirical and physical models might be interesting because the semi-empirical model simplifies the physical model with respect to the variation occurring in the angular sampling of the sensor and it might be a way to invert the physical model [25]. This again would favour a hybrid approach.

Laboratory goniometer measurements have the advantage of control of the illumination conditions and diffuse light can be neglected. On the other hand, the light source is at a limited distance from the target and as a result the light is not perfectly parallel as it is with the sun as illumination source [22]. In addition, when preparing to measure new effects such as single scattering albedo or scattering phase functions, very stable and controlled environments like in a laboratory are a requirement, in particular when using these methods in scaling approaches [31]. With respect to the target no wind effects are present, so the target will not move during the measurement sequence. Field goniometer measurements leave targets in their natural environment. Natural illumination by the sun is used. Disadvantage is that illumination can vary during the measurement sequence, particularly due to atmospheric effects. Also the diffuse illumination component cannot be neglected. Therefore, in the end scaling effects should be studied by linking research with laboratory goniometers with field goniometers and remote sensing observations [21].

## Figures and Tables

**Table 1.** Root mean square (RMS) differences of the repetitive reflectance measurements at nadir position of 18 days old lawn grass and watercress. The average RMS differences of measurement #2, #3 and #4 with #1 over the interval 500–1,900 nm are given.

**Target**	**Wavelength interval**	**Measurement comparison**		
		1–2	1–3	1–4
**lawn grass**	500–1,900 nm	0.00286	0.00183	0.00165
**watercress**	500–1,900 nm	0.00110	0.00182	0.00380
